# Cancer and the family: Variations by sex and race/ethnicity

**DOI:** 10.1002/cam4.6969

**Published:** 2024-02-01

**Authors:** Charlotte Asiedu, Nicole S. McKinney, Alliric I. Willis, Frances M. Lewis, Shannon Virtue, Adam Davey

**Affiliations:** ^1^ Department of Health Behavior and Nutrition Sciences University of Delaware Newark Delaware USA; ^2^ Department of Counseling and Behavioral Health Thomas Jefferson University Philadelphia Pennsylvania USA; ^3^ Department of Surgery, Surgical Oncology Thomas Jefferson University Philadelphia Pennsylvania USA; ^4^ Department of Child, Family, and Population Health Nursing University of Washington Seattle Washington USA; ^5^ Helen F Graham Cancer Center and Research Institute, Christiana Care Health System Newark Delaware USA

**Keywords:** adolescents, cancer survivors, children, family life cycle, kinship networks, parents

## Abstract

**Background:**

Cancer affects patients and their families, but few data are available on factors associated with diversity of family structures among patients with cancer. Family is a source of both support and responsibility that must be understood to support patients and their families.

**Methods:**

Pooled data (2004–2015) from the National Health Interview Study were used to compare characteristics of cancer survivors with and without minor children and differences by sex and race/ethnicity among survivors with minor children.

**Results:**

13.9% of cancer survivors have minor children in the household, and this experience is more likely for women and people who identify as other than non‐Hispanic White.

**Conclusion:**

There are considerable differences by sex and race/ethnicity in the characteristics of cancer survivors with minor children. Clinicians should make consideration of family circumstances a routine part of their history. Doing so will help to identify potential sources of support and responsibility that may affect adherence.

## Graphical abstract

Pooled data (2004–2015) from the National Health Interview Study were used to compare characteristics of cancer survivors with and without minor children and differences by sex and race/ethnicity among survivors with minor children. There are considerable differences by sex and race/ethnicity in the characteristics of cancer survivors with minor children. Clinicians should make consideration of family circumstances a routine part of their history to identify potential sources of support and responsibility that may affect adherence.

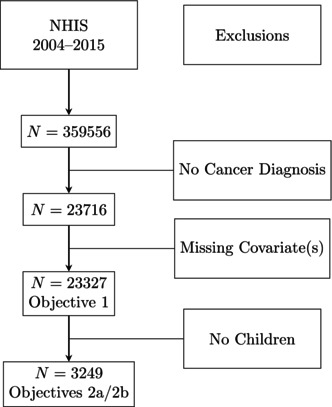

## 1 BACKGROUND

Cancer is marked by steep health gradients across numerous axes (i.e., disparities), particularly gender and race/ethnicity.^1^ At the same time, there is growing recognition that the effects of parental illness ripple through the entire family system, leading to an increased focus on the best ways to support patients and their families through the treatment process.^2^, ^3^

In cancer treatment, however, the focus rests squarely on the patient with cancer, to the point that local and national medical record and registry data contain exceptionally little information about family and household structure. Apart from women with breast cancer, for whom the collection of parity and gravidity information during history is standard, no information beyond marital status is typically collected or recorded. This makes very few resources available for researchers and clinicians for purposes of planning and identifying potential needs, responsibilities, and resources.

Recently, Weaver and colleagues used data from the National Health Interview Study (NHIS) to describe the characteristics of individuals with cancer and their own coresident minor children and estimated the prevalence of having minor children among cancer survivors to be approximately 14%.^4^ Using a different approach, Inhestern and colleagues conducted a systematic review and found that prevalence ranged between 14% and 24.7% depending on the sample structure.^5^ Building on their work, we replicate and extend Weaver's analyses to compare cancer survivors^6^ with and without family members under 18 in the household in order to consider differences by sex and race/ethnicity. This is critically important because many cancers solely or predominantly affect men or women, many types of cancer are more common at some ages than others, and some cancers are more and less common in some racial and ethnic groups.^7^ Likewise, the family circumstances of men and women and across racial and ethnic groups vary widely in ways that can affect their ability to adhere to clinical recommendations through support or responsibilities.

The objectives of the current study were: (1) to characterize systematic differences between cancer survivors with and without minor children in the household and (2) to elaborate on the characteristics of cancer survivors with minor children in the household by: (a) sex and (b) race/ethnicity.

## 2 INTRODUCTION

United States cancer statistics for 2019 reported that approximately 18% of cancer cases affected 15‐ to 54‐year‐old, the ages most individuals spend having and rearing their children, and thus greater likelihood for these individuals to have minors under their care.^8^

Cancer health disparities are prevalent in the U.S. and are most pronounced between Blacks and Whites in the U.S. Nationally representative data from SEER demonstrate that Blacks have a cancer incidence that is 3.5% lower than Whites but have a cancer death rate that is 12.4% higher than for Whites.^9^ This disparity is particularly noteworthy and reflects historical factors particularly unique to the U.S. This contrasts with data demonstrating that Hispanics and Asians in the U.S. have a lower incidence and lower cancer death rate than Whites.^9^ Healthcare system factors, including mistrust of providers, as well as reduced access to insurance and quality providers. Socioeconomic gradients can affect access to health care through reduced access to transportation, job flexibility, and other challenges to screening and preventive care as well as treatment adherence.^10^ Several modifiable and nonmodifiable risk factors also contribute to health disparities including environmental and occupational exposures and, in some cases, genetic ancestry.^10^

Differences in cancer incidence and mortality transcend gender. Black women have lower incidence, but higher cancer death rate, than White women.^9^ Breast cancer‐specific mortality is higher among African American, American Indian/Alaskan Native and Hispanic/Latina women than non‐Hispanic Whites. Prostate cancer is the most commonly occurring cancer among men and also constitutes the largest of all cancer differences. African American men have a 78% higher incidence rate than non‐Hispanic White men.^10^ They are also more likely to be diagnosed at a younger age but also to present with more advanced and aggressive disease, resulting in a mortality rate that is 2.3 times higher than for non‐Hispanic White men.^10^ Similar trends exist across cancers for both genders including colon, pancreas, and lung cancers. Racial disparities in cancer‐related mortality transcend stage; Black patients have higher cancer death rates at every stage across multiple cancers including breast, uterus, colon, lung, stomach, and prostate cancers.^9^ Especially impactful to families affected by cancer is that medically related issues including medical bills and loss of income due to illness are the cause of bankruptcy claims in the U.S.^11^

A cancer diagnosis does not affect a patient in isolation, but ripples through the family affecting relationships with spouses and offspring most acutely. There is growing recognition that adolescents wish for greater information about their parent's cancer and increased peer support.^12^ Adolescents and young adults are particularly vulnerable to negative consequences associated with a parent's illness including anxiety, depression, and suicidal ideation.^13^ Currently, in cancer diagnosis and treatment, the focus is individually focused to the extent that few data are currently available to understand the family and living arrangements of individuals with cancer. This is important because family can serve both as a source of support, encouraging adherence, such as through direct support, instrumental support, financial contributions, and indirectly such as assisting with childcare responsibilities. Family is also a source of responsibility that needs to be fully considered in treatment planning, such as around school and activity schedules of minor children, and the responsibilities inherent to caring for minor children and being a spouse or partner.^14^ The lack of representative data about family structure of individuals with cancer also makes study planning a challenge. Questions as elemental as the proportion of men and women diagnosed with cancer at the same time that they also have minors in the household is unknown, representing a significant knowledge gap.^15^

The increased financial and emotional burden parents must deal with affects their presence in their children's lives, their styles of parenting, and even the quality of life they can offer their children. These children may have to witness sudden changes in their parents, watch them suffer, or, in some cases, face the possibility of death. Cancer treatments, such as chemotherapy and radiation, can cause fatigue and make it difficult for parents to keep up with their usual responsibilities.^8^ Additionally, parents may be unable to provide the same level of emotional support and attention to their children as they ordinarily would.^16^

There is growing evidence that parental cancer increases the risk of developmental and psychosocial problems in children. It can have a negative impact on the mental and physical health of children, as well as their academic performance.^8^, ^17^ Children may also be more prone to behavioral and emotional issues, as well as having trouble establishing and maintaining relationships. Many studies have focused on the immediate impacts of parental cancer on children, but there is little research on how effects persist over time.

Considering cancer survivors specifically, there has been little research into how they cope with childrearing, given the difficulties they might have faced and the possibility of cancer recurrence. There is also a lack of studies focused on the diversity of families among cancer survivors in terms of race, marital dynamics, level of support, and other key characteristics. Clearly, cancer disproportionately affects certain populations, such as low‐income families and minority groups, but most studies have not accounted for these factors.^18^ It is important to understand how the experience of having a parent with cancer may differ for children from different backgrounds and how these differences may impact their development.^16^, ^19^, ^20^, ^21^

Gathering data on parental cancer across different populations can provide currently unavailable but potentially valuable information for researchers, healthcare professionals, and policymakers. It can help identify patterns and trends in cancer incidence and mortality among different groups and disparities in these groups.^22^ By gathering data on parental cancer in these populations, researchers can identify the factors that contribute to these disparities and ultimately develop interventions to address them.^10^

## 3 METHODS

### 3.1 Study design

This study employed secondary analysis of 12 years of nationally representative pooled cross‐sectional data from 2004 to 2015, the largest number of contiguous waves of NHIS data since 2004 that could be combined without being affected by changes in weighting procedures and study design.

### 3.2 Variables

*Sex* was coded male or female. *Age group* was coded as 18–39 years, 40–49 years, 50–64 years, and 65 years and older. *Marital status* was coded as married, widowed/divorced/separated, never married, and living with a partner. *Educational attainment* was coded as less than high school, completed high school/GED, some college, a bachelor's degree, or more education. *Race/ethnicity* was coded as White, non‐Hispanic; Hispanic; Black, non‐Hispanic; and Other, non‐Hispanic. *Number of family members under 18 in the household* was coded as 0, 1, 2, 3, 4, or ≥5, which was based upon one of the constructed variables capturing aspects of household structure. Specifically, we used the FM_KIDS variable distributed with the NHIS. This variable was selected in order to ensure that we captured living arrangements such as custodial grandparenting so that we would not inadvertently exclude older patients from consideration. *Cancer sites* were coded as cervix, breast, uterus, melanoma, ovary, thyroid, leukemia/lymphoma, colon, testicle, and other sites. *Time since cancer diagnosis* was coded as within 1 year, 2–5 years, 6–10 years, and 11 or more years. *Age at first cancer diagnosis* was coded as under 20 years, 20–29 years, 30–49 years, and 40 years.

### 3.3 Statistical methods

NHIS uses a complex survey sampling design that requires adjustment during analysis to obtain statistically valid parameter estimates and standard errors. Consistent with recommendations by the data source, sampling weights were divided by the number of interview waves pooled to provide a denominator representing the average population size during the period considered. Survey‐adjusted binary (minors in household and sex) or multinomial (race/ethnicity) logistic regression models were estimated as an omnibus test to preserve the overall Type I error rate in subsequent comparisons. Because prevalence of many cancer types differed so widely between men and women, they had to be excluded from the multivariable model predicting differences between men and women. Survey‐adjusted means and proportions were estimated for all study variables across sex and race/ethnicity categories. Survey‐adjusted cross‐tabulations were estimated with associations evaluated via *F*‐tests, with significant effects followed up with survey‐adjusted binary or multinomial logistic regression models for purposes of interpretation. All analyses were performed using Stata 18.0.^23^

## 4 RESULTS

### 4.1 Participants

**FIGURE 1:**
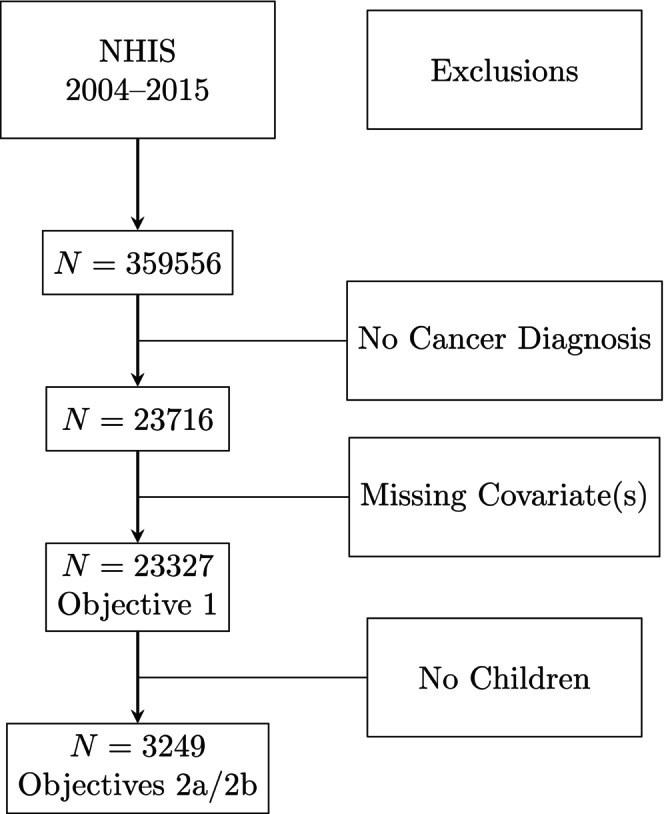
STROBE diagram for analytic samples.

Overall, 359,556 individuals participated in one of the NHIS waves considered. Of these, 23,716 (6.6%) reported a cancer diagnosis. Among those diagnosed with cancer, 3291 (13.9%) had a coresident minor child and 20,425 (86.1%) did not. Data were missing for a small subset of cases on marital status, educational attainment, race/ethnicity, and timing of diagnosis, yielding an overall listwise complete sample size across all study variables of 23,327 individuals, of whom 20,078 had cancer but no children and 3249 had cancer and a minor child. Cases affected by incomplete data were too few to affect findings in a meaningful way.^24^ Thus, final analytic sample size was 23,327 for Objective 1 and was 3249 for Objectives 2a and 2b (Figure 1).

### 4.2 Descriptive results

Cancer survivors were 40.3% men and 59.7% women, and 13.9% reported having a minor child. Non‐Hispanic Whites comprised 82.9% of cancer survivors; 5.8% were Hispanic, 7.5% were non‐Hispanic Black, and 3.8% reported being non‐Hispanic of Other race. Some 8.5% were aged 18–39 years, 10.7% were 40–49 years, 30.6% were 50–64 years, and 50.2% were 65+. A majority (58.6%) of survivors were currently married, with 30.0% being divorced, separated, or widowed, 7.2% never married, and 4.2% living with a partner. Approximately 9.0% of cancer survivors had less than high school education; 24.3% had completed high school or a GED; 30.7% of survivors had some college education, and 36.0% had completed at least a bachelor's degree.

### 4.3 Objective 1: Comparison of cancer survivors with and without minor children

Characteristics of cancer survivors with (*n* = 3249) and without (*n* = 20,078) minor children are shown in Table 1. The overall multivariable model was statistically significant (F (29, 253) = 76.48, *p* < 0.0001), and so we proceed to interpretation of the individual characteristics. As can be seen, survivors with children were more likely to be women (72.3% vs. 57.2%, F (1.0, 281.0) = 163.97, *p* < 0.0001). Survivors with children were considerably younger than those without minor children (F (2.9, 819.0) = 1150.80, *p* < 0.0001) across all categories.

**TABLE 1:** Characteristics of cancer survivors with and without minor children (*N* = 23,327).

Variable	No minor children		Minor children		*p*‐Value
	Wtd %	95% CI	Wtd %	95% CI	
Sex					
Male	42.7%	[41.9%–43.6%]	28.3%	[26.2%–30.3%]	<0.0001
Female	57.3%	[56.4%–58.1%]	71.7%	[69.7%–73.8%]	
Age group					
18–39	4.0%	[3.7%–4.4%]	31.3%	[29.4%–33.2%]	<0.0001
40–49	6.5%	[6.0%–7.0%]	32.2%	[29.9%–34.6%]	
50–64	31.7%	[30.8%–32.6%]	25.2%	[23.1%–27.2%]	
65+	57.7%	[56.7%–58.8%]	11.3%	[9.8%–12.7%]	
Marital status					
Married	57.7%	[56.7%–58.7%]	63.3%	[61.0%–65.7%]	<0.0001
Wid/div/sep	31.4%	[30.5%–32.3%]	22.6%	[20.7%–24.4%]	
Never married	6.9%	[6.5%–7.4%]	8.3%	[7.2%–9.4%]	
Live with partner	4.0%	[3.6%–4.4%]	5.8%	[4.7%–6.9%]	
Education					
<High school	9.2%	[8.6%–9.7%]	6.5%	[5.6%–7.3%]	<0.0001
High school/GED	24.5%	[23.7%–25.3%]	22.0%	[20.0%–23.9%]	
Some college	30.0%	[29.1%–31.0%]	34.7%	[32.4%–37.1%]	
≥Bachelor's	36.3%	[35.2%–37.4%]	36.9%	[34.3%–39.4%]	
Race/ethnicity					
White, non‐Hispanic	84.5%	[83.9%–85.2%]	73.6%	[71.6%–75.5%]	<0.0001
Hispanic	4.9%	[4.5%–5.2%]	11.0%	[9.7%–12.3%]	
Black, non‐Hispanic	7.1%	[6.5%–7.6%]	10.0%	[8.6%–11.3%]	
Other, non‐Hispanic	3.6%	[3.2%–3.9%]	5.5%	[4.3%–6.6%]	
Cervix	6.7%	[6.2%–7.2%]	22.1%	[20.3%–23.9%]	<0.0001
Breast	23.6%	[22.8%–24.3%]	15.9%	[14.4%–17.4%]	<0.0001
Uterus	5.7%	[5.3%–6.1%]	6.0%	[4.9%–7.0%]	0.3407
Melanoma	10.2%	[9.6%–10.7%]	9.7%	[8.3%–11.2%]	0.5388
Ovary	2.9%	[2.6%–3.2%]	4.5%	[3.6%–5.4%]	0.0001
Thyroid	2.9%	[2.6%–3.2%]	6.0%	[4.9%–7.1%]	<0.0001
Leuk/lymph	5.8%	[5.4%–6.3%]	7.0%	[5.6%–8.3%]	0.0645
Colon	8.8%	[8.3%–9.3%]	4.3%	[3.4%–5.3%]	<0.0001
Testis	1.1%	[0.8%–1.3%]	2.0%	[1.4%–2.7%]	0.0006
Other	42.6%	[41.7%–43.4%]	31.1%	[29.0%–33.2%]	<0.0001
Time since Dx					
0–1	13.7%	[13.0%–14.4%]	16.4%	[14.8%–17.9%]	<0.0001
2–5	25.6%	[24.8%–26.4%]	29.3%	[27.0%–31.6%]	
6–10	20.6%	[19.9%–21.3%]	21.0%	[19.2%–22.7%]	
11+	40.2%	[39.2%–41.1%]	33.4%	[31.0%–35.7%]	
Age at first Dx					
<20	4.0%	[3.6%–4.5%]	11.3%	[9.8%–12.9%]	<0.0001
20–29	6.4%	[6.0%–6.9%]	23.5%	[21.7%–25.4%]	
30–39	9.1%	[8.6%–9.7%]	22.5%	[20.5%–24.5%]	
≥40	80.4%	[79.6%–81.2%]	42.7%	[40.2%–45.1%]	

While a majority (57.7%) of survivors without minor children were aged 65+, only 11.4% of survivors with minor children were aged 65+. Cancer survivors with children were more likely to be married (63.3% vs. 57.7%) or partnered (5.7% vs. 4.0%), and less likely to be divorced (22.7% vs. 31.4%; F (2.8, 791.4) = 24.72, *p* < 0.0001). Cancer survivors with minor children had higher levels of educational attainment (F (2.8, 781.1) = 10.80, *p* < 0.0001) across all categories. There were also differences in race/ethnicity by parental status such that survivors with children were less likely to be non‐Hispanic White (73.7% vs. 84.7%, F (2.9, 807.3) = 61.27, *p* < 0.0001).

There were no differences by parental status in cancers of the uterus (F (1.0, 281.0) = 0.91, *p* = 0.3407), melanoma (F (1.0, 281.0) = 0.38, *p* = 0.5388), or leukemia/lymphoma (F (1.0, 281.0) = 3.44, *p* = 0.0645). Cancers of the cervix (F (1.0, 281.0) = 567.86, *p* < 0.0001), ovary (F (1.0, 281.0) = 15.96, *p* = 0.0001), thyroid (F (1.0, 281.0) = 48.28, *p* < 0.0001), and testis (F (1.0, 281.0) = 12.14, *p* = 0.0006) were all more common among survivors with children, whereas cancers of the breast (F (1.0, 281.0) = 66.10, *p* < 0.0001), colon (F (1.0, 281.0) = 43.57, *p* < 0.0001), and other sites (F (1.0, 281.0) = 107.87, *p* < 0.0001) were less common among survivors with children. Survivors with children were on average closer to their first diagnosis (F (2.8, 780.5) = 13.46, *p* < 0.0001) and younger at age of first diagnosis (F (2.9, 812.5) = 413.01, *p* < 0.0001).

Below, we elaborate these generalizations to consider diversity in family structure among cancer survivors with minor children in the household by sex and race/ethnicity.

### 4.4 Objective 2a: Comparison of cancer survivors with children by sex

Characteristics of men and women cancer survivors with minor children are presented in Table 2. The overall multivariable model, exclusive of cancer location indicators, was statistically significant (F (18, 262) = 25.57, *p* < 0.0001), and so we proceed to interpretation of the individual characteristics. Among this group, men are older than women, on average (F (2.9, 812.0) = 48.13, *p* < 0.0001) across all age categories. Men are more likely to be married than women (79.7% vs. 57.0%) and women are more likely than men to be divorced/widowed/separated (26.9% vs. 11.8%), never married (9.9% vs. 4.3%), or cohabiting (6.2% vs. 4.3%, F (2.9, 795.4) = 27.51, *p* < 0.0001). Men had higher educational attainment than women, on average, with 44.2% of men having at least a bachelor's degree compared with 33.3% of women (F (2.9, 808.9) = 7.95, *p* < 0.0001). There were no sex differences by race/ethnicity (F (2.9, 805.2) = 1.79, *p* = 0.1501) or number of children (F (3.9, 1080.2) = 1.36, *p* = 0.2477).

**TABLE 2:** Cancer survivors with children by sex (*N* = 3249).

	Men		Women		*p*‐Value
	Wtd %	95% CI	Wtd %	95% CI	
Age group					
18–39	15.2%	[12.1%–18.3%]	37.6%	[35.4%–39.9%]	<0.0001
40–49	29.0%	[24.7%–33.3%]	33.5%	[30.9%–36.2%]	
50–64	36.0%	[31.6%–40.4%]	20.9%	[18.6%–23.3%]	
65+	19.8%	[16.5%–23.2%]	7.9%	[6.5%–9.3%]	
Marital status					
Married	79.9%	[76.0%–83.7%]	56.8%	[54.0%–59.6%]	<0.0001
Wid/div/sep	11.7%	[8.8%–14.5%]	26.9%	[24.6%–29.2%]	
Never married	4.4%	[2.3%–6.6%]	9.9%	[8.6%–11.2%]	
Live with partner	4.1%	[2.3%–5.8%]	6.5%	[5.2%–7.8%]	
Education					
<High school	5.7%	[4.1%–7.3%]	6.8%	[5.7%–7.8%]	<0.0001
High school/GED	20.0%	[16.5%–23.5%]	22.8%	[20.5%–25.0%]	
Some college	29.3%	[25.1%–33.4%]	36.9%	[34.1%–39.6%]	
≥Bachelor's	45.1%	[40.6%–49.6%]	33.6%	[30.8%–36.5%]	
Race/ethnicity					
White, non‐Hispanic	73.0%	[69.4%–76.7%]	73.8%	[71.6%–75.9%]	0.1501
Hispanic	9.5%	[7.3%–11.6%]	11.6%	[10.2%–13.0%]	
Black, non‐Hispanic	11.5%	[8.7%–14.4%]	9.3%	[8.0%–10.7%]	
Other, non‐Hispanic	6.0%	[4.2%–7.8%]	5.3%	[3.9%–6.6%]	
Number of children					
1	49.6%	[45.0%–54.2%]	46.6%	[43.9%–49.3%]	0.2477
2	34.0%	[29.6%–38.4%]	34.0%	[31.5%–36.5%]	
3	11.1%	[8.2%–14.0%]	13.2%	[11.6%–14.7%]	
4	4.5%	[2.6%–6.4%]	4.3%	[3.2%–5.4%]	
5+	0.8%	[0.1%–1.6%]	1.9%	[1.2%–2.6%]	
Cervix	0.0%	–	30.8%	[28.5%–33.2%]	–
Breast	0.4%	[0.0%–0.8%]	22.0%	[20.0%–24.0%]	<0.0001
Uterus	0.0%	–	8.3%	[6.9%–9.8%]	–
Melanoma	15.5%	[12.0%–19.0%]	7.4%	[6.0%–8.9%]	<0.0001
Ovary	0.0%	–	6.3%	[5.0%–7.5%]	–
Thyroid	3.1%	[1.5%–4.6%]	7.1%	[5.8%–8.5%]	0.0019
Leuk/lymph	13.2%	[9.8%–16.5%]	4.5%	[3.4%–5.6%]	<0.0001
Colon	6.9%	[4.9%–8.9%]	3.3%	[2.3%–4.4%]	0.0002
Testis	7.2%	[4.9%–9.6%]	0.0%	–	–
Other	62.3%	[58.1%–66.6%]	18.8%	[16.8%–20.9%]	<0.0001
Time since Dx					
0–1	19.4%	[15.9%–22.9%]	15.2%	[13.3%–17.0%]	<0.0001
2–5	35.6%	[31.2%–40.1%]	26.8%	[24.4%–29.2%]	
6–10	16.2%	[12.9%–19.6%]	22.8%	[20.8%–24.9%]	
11+	28.7%	[24.3%–33.2%]	35.2%	[32.6%–37.8%]	
Age at first Dx					
<20	8.7%	[5.9%–11.4%]	12.4%	[10.6%–14.1%]	<0.0001
20–29	11.7%	[8.8%–14.7%]	28.2%	[25.8%–30.5%]	
30–39	16.4%	[13.1%–19.7%]	24.8%	[22.5%–27.2%]	
≥40	63.1%	[58.8%–67.5%]	34.6%	[31.8%–37.4%]	

Among cancer survivors with minor children, there were differences between men and women for every type of cancer considered. Clearly, cancers of the cervix, uterus, and ovaries were limited to women and cancer of the testis was limited to men, with breast cancer affecting 0.4% of men and 22.1% of women (F (1.0, 279.0) = 237.30, *p* < 0.0001). Beyond that, however, thyroid cancer was more common among women than men (6.9% vs. 3.1%, F (1.0, 279.0) = 9.79, *p* = 0.0019), whereas melanoma (F (1.0, 279.0) = 29.06, *p* < 0.0001), leukemia/lymphoma (F (1.0, 279.0) = 46.37, *p* < 0.0001), colon (F (1.0, 279.0) = 14.17, *p* = 0.0002), and other sites (F (1.0, 279.0) = 406.98, *p* < 0.0001) were all significantly more common among men than women. Men with minor children had shorter times since diagnosis, on average, than women (F (2.9, 810.4) = 9.57, *p* < 0.0001), and women with minor children had earlier ages of first diagnosis than men (F (3.0, 830.0) = 43.37, *p* < 0.0001).

### 4.5 Objective 2b: Comparison of cancer survivors with children by race/ethnicity

Table 3 presents characteristics of cancer survivors with minor children by race/ethnicity category. The overall multivariable model was statistically significant (F (78, 202) = 6.50, *p* < 0.0001), and so we proceed to interpretation of the individual characteristics. There were no differences by sex (F (2.9, 805.2) = 1.79, *p* = 0.1501). However, there were some age differences (F (8.4, 2353.3) = 6.09, *p* < 0.0001) such that Hispanics with minor children were somewhat more likely to be in the 18–39 age group compared with other ethnicities, and Whites with minor children were somewhat less likely to be in the 65+ age group. There were also differences in marital status (F (6.9, 1925.2) = 14.02, *p* < 0.0001), such that non‐Hispanic Blacks were less likely to be married and more likely to be widowed/divorced, separated, or never married, compared with other groups.

**TABLE 3:** Cancer survivors with children by race/ethnicity (*N* = 2959).

	White, non‐Hispanic		Hispanic		Black, non‐Hispanic		Other, non‐Hispanic		*p*‐Value
	Wtd %	95% CI	Wtd %	95% CI	Wtd %	95% CI	Wtd %	95% CI	
Sex									
Male	28.1%	[25.6%–30.6%]	24.2%	[19.7%–28.8%]	32.7%	[26.5%–38.9%]	31.0%	[22.6%–39.4%]	0.1501
Female	71.9%	[69.4%–74.4%]	75.8%	[71.2%–80.3%]	67.3%	[61.1%–73.5%]	69.0%	[60.6%–77.4%]	
Age group									
18–39	31.1%	[28.7%–33.5%]	35.9%	[30.6%–41.3%]	29.3%	[23.5%–35.1%]	28.0%	[20.3%–35.7%]	<0.0001
40–49	34.7%	[31.9%–37.5%]	29.5%	[24.3%–34.8%]	20.1%	[14.7%–25.5%]	26.4%	[18.0%–34.8%]	
50–64	25.3%	[22.8%–27.7%]	19.4%	[15.0%–23.8%]	29.0%	[23.3%–34.6%]	28.7%	[19.4%–38.1%]	
65+	8.9%	[7.3%–10.5%]	15.2%	[10.7%–19.6%]	21.6%	[16.6%–26.6%]	16.9%	[9.6%–24.2%]	
Marital status									
Married	67.4%	[64.6%–70.3%]	58.5%	[53.1%–63.9%]	39.7%	[32.8%–46.6%]	60.3%	[49.8%–70.7%]	<0.0001
Wid/div/sep	21.2%	[19.0%–23.4%]	23.8%	[19.2%–28.3%]	31.4%	[25.3%–37.4%]	23.0%	[16.1%–29.9%]	
Never married	5.4%	[4.3%–6.6%]	13.3%	[9.7%–17.0%]	21.3%	[16.6%–26.0%]	13.4%	[4.2%–22.6%]	
Live with partner	5.9%	[4.5%–7.3%]	4.4%	[2.2%–6.6%]	7.6%	[4.3%–10.9%]	3.3%	[0.7%–6.0%]	
Education									
<High school	4.7%	[3.8%–5.6%]	16.2%	[12.3%–20.0%]	10.4%	[7.6%–13.2%]	3.5%	[1.0%–6.1%]	<0.0001
High school/GED	21.6%	[19.2%–23.9%]	26.3%	[21.0%–31.6%]	21.3%	[16.4%–26.2%]	20.1%	[11.0%–29.1%]	
Some college	33.1%	[30.3%–35.9%]	38.9%	[32.5%–45.2%]	45.2%	[39.5%–50.9%]	29.2%	[20.3%–38.0%]	
≥Bachelor's	40.7%	[37.4%–43.9%]	18.7%	[14.3%–23.1%]	23.1%	[18.2%–28.1%]	47.2%	[37.0%–57.4%]	
Number of children									
1	47.6%	[44.7%–50.5%]	43.7%	[38.8%–48.6%]	49.8%	[43.9%–55.8%]	49.0%	[39.5%–58.5%]	0.0284
2	34.6%	[31.8%–37.4%]	32.0%	[27.2%–36.7%]	31.7%	[25.5%–37.8%]	34.9%	[25.3%–44.5%]	
3	12.1%	[10.4%–13.9%]	18.8%	[14.3%–23.3%]	11.5%	[8.0%–15.0%]	7.9%	[3.8%–11.9%]	
4	4.1%	[3.0%–5.1%]	4.5%	[2.3%–6.6%]	4.8%	[1.3%–8.3%]	7.5%	[2.7%–12.3%]	
5+	1.6%	[1.0%–2.3%]	1.1%	[0.2%–1.9%]	2.2%	[0.3%–4.2%]	0.7%	[0.0%–1.8%]	
Cervix	23.3%	[21.1%–25.4%]	20.1%	[15.8%–24.4%]	19.9%	[14.9%–24.8%]	14.4%	[8.3%–20.5%]	0.0925
Breast	14.7%	[12.9%–16.6%]	20.5%	[16.1%–24.9%]	18.1%	[13.2%–23.0%]	18.3%	[12.3%–24.2%]	0.0962
Uterus	5.7%	[4.4%–7.0%]	7.6%	[5.0%–10.3%]	5.4%	[3.0%–7.8%]	6.7%	[2.6%–10.7%]	0.5797
Melanoma	12.2%	[10.4%–14.1%]	2.0%	[0.8%–3.3%]	1.0%	[0.0%–2.1%]	7.2%	[2.0%–12.4%]	<0.0001
Ovary	3.9%	[2.9%–5.0%]	7.0%	[4.0%–9.9%]	5.4%	[2.6%–8.2%]	5.4%	[1.7%–9.1%]	0.0405
Thyroid	5.8%	[4.7%–7.0%]	6.4%	[3.4%–9.4%]	3.9%	[1.5%–6.2%]	10.9%	[3.3%–18.5%]	0.1390
Leuk/lymph	7.2%	[5.6%–8.7%]	7.5%	[4.1%–10.8%]	6.1%	[3.0%–9.1%]	4.5%	[0.5%–8.6%]	0.8052
Colon	3.9%	[2.8%–5.0%]	2.1%	[0.7%–3.5%]	7.7%	[4.2%–11.1%]	8.7%	[4.4%–13.0%]	0.0022
Testis	2.4%	[1.5%–3.3%]	1.1%	[0.1%–2.1%]	0.4%	[0.0%–1.1%]	2.4%	[0.0%–5.2%]	0.0841
Other	29.4%	[27.0%–31.9%]	35.2%	[30.2%–40.2%]	39.4%	[33.0%–45.7%]	30.8%	[22.0%–39.6%]	0.0025
Time since Dx									
0–1	15.3%	[13.4%–17.2%]	18.7%	[15.0%–22.4%]	19.6%	[14.7%–24.5%]	20.0%	[12.3%–27.6%]	0.0102
2–5	28.6%	[25.8%–31.3%]	33.2%	[28.1%–38.3%]	30.0%	[23.6%–36.3%]	29.6%	[20.9%–38.3%]	
6–10	20.3%	[18.3%–22.3%]	20.7%	[16.5%–24.8%]	24.0%	[18.9%–29.2%]	24.9%	[15.9%–33.9%]	
11+	35.8%	[32.9%–38.7%]	27.4%	[22.0%–32.8%]	26.4%	[21.3%–31.5%]	25.6%	[17.9%–33.3%]	
Age at first Dx									
<20	12.3%	[10.4%–14.2%]	7.8%	[4.4%–11.1%]	8.4%	[5.1%–11.6%]	11.0%	[4.1%–17.9%]	0.0309
20–29	24.0%	[21.7%–26.3%]	25.3%	[20.4%–30.3%]	20.2%	[15.2%–25.2%]	19.5%	[12.6%–26.4%]	
30–39	22.6%	[20.2%–25.0%]	25.2%	[20.6%–29.8%]	18.6%	[13.3%–24.0%]	22.0%	[14.1%–29.9%]	
≥40	41.1%	[38.2%–43.9%]	41.7%	[36.3%–47.1%]	52.8%	[46.3%–59.2%]	47.5%	[37.9%–57.2%]	

Educational attainment also differed significantly across groups (F (8.2, 2274.4) = 13.97, *p* < 0.0001) with Hispanic and non‐Hispanic Blacks less likely than non‐Hispanic Whites to have at least a bachelor's degree and more likely to have less than high school education. There were small differences in the number of children (F (10.1, 2831.5) = 2.01, *p* = 0.0284) across race/ethnicity groups, such that Hispanics were slightly less likely to have just one child and were slightly more likely to have 3 children, whereas other non‐Hispanic parents were slightly more likely to have 2 children.

There were no differences by race/ethnicity in cervical (F (3.0, 830.8) = 2.15, *p* = 0.0925), breast (F (2.9, 817.4) = 2.13, *p* = 0.0962), uterine (F (2.8, 779.6) = 0.64, *p* = 0.5797), thyroid (F (2.7, 749.1) = 1.87, *p* = 0.1390), leukemia/lymphoma (F (3.0, 830.7) = 0.33, *p* = 0.8052), or testicular cancers (F (2.9, 798.5) = 2.25, *p* = 0.0841). There were differences by race in rates of ovarian cancer (F (3.0, 831.7) = 2.78, *p* = 0.0405), such that rates were highest among Hispanics (7.2%) and lowest among non‐Hispanic Whites (3.9%). Melanomas were considerably less common among Hispanic and non‐Hispanic Black groups (F (2.7, 744.7) = 19.71, *p* < 0.0001). Colon cancer was more common among non‐Hispanic Black and Other groups (F (2.9, 807.6) = 4.99, *p* = 0.0022). Cancers in other sites were most common among non‐Hispanic Blacks (F (2.9, 818.5) = 4.88, *p* = 0.0025). On average, non‐Hispanic Whites were somewhat more likely to be further from diagnosis than other groups (F (8.3, 2314.2) = 2.48, *p* = 0.0102), and Hispanics and non‐Hispanic Blacks were less likely to be diagnosed before age 20 and non‐Hispanic Blacks were more likely to be diagnosed after age 40 (F (8.4, 2340.0) = 2.09, *p* = 0.0309).

## 5 DISCUSSION

We presented results comparing cancer survivors with and without minor children (objective 1) and provided comparisons between men and women (objective 2a) and across racial/ethnic categories (objective 2b). Results indicate that the probability of a person with cancer also having minor children in the household is common and varies considerably by sex and race/ethnicity.

Overall, nearly 1 in 7 cancer survivors lives with a minor child. Cancer survivors with minor children were more likely to be women and identify as other than non‐Hispanic White, to be closer to their first diagnosis and to have been younger at age of first diagnosis. Women were much more likely than men to have cancer diagnosis and minor children in the household. Women cancer survivors with children were much more likely than men to be unmarried. Hispanic cancer survivors with children were more likely to be younger and have lower educational attainment. Having coresident minor children was more likely for individuals diagnosed with cancers of cervix, breast, ovary, thyroid, and testis, but less likely to be diagnosed with colon cancer.

These overall differences mask considerable variability in the experiences of men and women. Compared to men with minor children, women with minor children were, on average, younger, less likely to be married or partnered, and to have lower educational attainment. Having minor children was more common for women than men for breast and thyroid cancer, but less likely for women than men for diagnoses of melanoma, leukemia/lymphoma, colon, testis, and other cancer sites. Women with minor children were further from their first diagnosis than men, but first diagnoses are earlier for women than men with minor children.

There were also considerable variations in family structure by race/ethnicity. Specifically, Hispanics were more likely to be in the youngest age group, compared with other groups. In contrast, Black, non‐Hispanic survivors with minor children were more likely to be in the oldest age category (21.6% 65+ for non‐Hispanic Black survivors, compared with 8.9% among non‐Hispanic White survivors). Compared with non‐Hispanic White survivors, non‐Hispanic Black survivors were less likely to be married (67.4% compared with 39.7%). Educational attainment was lower among Hispanic and non‐Hispanic Black survivors compared with individuals reporting non‐Hispanic White and Other race, with 18.7% and 23.1% of individuals in the former two groups reporting some college education compared with 40.7% and 47.2% of individuals in the latter two groups, respectively. Compared with other groups, non‐Hispanic Whites with minor children were more likely to be further from their date of first diagnosis, but also more likely to have received it at younger ages.

By virtue of its nationally representative survey design, the 23,327 cancer survivors considered here correspond with an average of approximately 13,624,188 U.S. cancer survivors in each year considered. Likewise, the subset of 3249 cancer survivors with minor children corresponds with an average of approximately 2,216,519 families nationally in each year considered. The data reported can also help in the process of sample size planning, including estimating the number of individuals who are likely to meet eligibility criteria and the number of clinical sites needed to support an intervention.

Because analyses are based on a representative sample of the United States population for each year that the study was conducted, we expect that the results should largely generalize to the population of cancer survivors with minor children in the household. However, limitations include the fact that the NHIS includes no information about noncustodial parental experiences and our results would average any secular trends. Equally importantly, the broad nature of the NHIS prevents collection of relevant details related to diagnoses, treatment, and prognosis. Even age at diagnosis and time since diagnosis are collected with variable granularity.

The current research has applications for researchers, clinicians, patients, and their families. Data on family structure of patients with cancer are useful for researchers to generate realistic expectations with regard to screening. For example, among the patients with colon cancer seen annually at a clinical, how many would be expected to have childcare responsibilities? Likewise, a researcher interested in understanding the experience of mothers with cancer who are at least 2 years from initial diagnosis might wish to anticipate the distribution of cases across cancer types to prioritize support group attendance for recruitment efforts. Overall, having access to realistic data on potential sources of family support and obligation could be useful for understanding the experience of living with cancer. For this reason, there is a potential value to including the kind of information about family relationships and responsibilities that are vital for supporting all patients through the treatment process alongside existing registry data fields.

To date, several intervention studies focused on individuals with cancer and minor children in the home, typically further restricted to children in specific age ranges. Several psychosocial interventions focus on quality of life and communication outcomes, one in a hematologic cancer population,^25^ one including patients with stages I–III cancers and children aged 5–14 years,^26^ and two with cancer staging not described and children aged 3–18^27^ and 5–12 years,^28^ respectively. Another set of studies by Lewis and colleagues address cancer parenting among individuals with stage IV cancers and children aged 5–17 years,^29^ or stages 0–III cancers and children aged 5–12^30^ and a multi‐state study for children aged 8–12 years,^30^ respectively. There are also examples of culturally adapted interventions. At least two programs have been developed for African American families experiencing cancer stages I–III with children aged 10–18^31^ and with stages 0–III cancers and children aged 11–21.^7^ A telephone‐based cancer education program directed at Hispanic mothers included individuals with cancer stages 0–III and children aged 5–17 years.^29^ Finally, at least one intervention selects based on characteristics of both parents (high potentcy cannabis use among individuals) with stage IV cancers and adolescents aged 5–7 years demonstrating aggressive behavior.^32^

For clinicians, routine collection of information about parental status, presence and ages of children in the home, as well as ongoing familial responsibilities could facilitate and enhance discussion of potential supports and barriers to treatment adherence for patients of all ages. Families represent essential stakeholders to consider in the process of clinical decision‐making. It is also important to remember that the presence of a minor child in the household is not evenly distributed across patients with cancer. Factors including sex, race–ethnicity, marital and parental status combine in ways that make individuals with some characteristics much more likely to also have coresident minors. Clinicians may also be able provide suggestions and advice on navigating conversations with family members about their diagnosis, treatment, and prognosis.

Finally, it is our hope that patients and families can ultimately benefit from the downstream outcomes associated with greater understanding of families and illness. Cancer care remains very firmly individually focused. Ultimately, patients and their families can benefit from the greater opportunities for psycho‐oncological interventions to support them through the medical treatment while positioning them well for the highest possible quality of life, moving forward.

## AUTHOR CONTRIBUTIONS

**Charlotte Asiedu:** Conceptualization (equal); formal analysis (equal); methodology (equal); software (equal); writing – original draft (lead); writing – review and editing (equal). **Nicole S. McKinney:** Project administration (equal); writing – review and editing (equal). **Alliric I. Willis:** Conceptualization (equal); writing – original draft (equal); writing – review and editing (equal). **Frances M. Lewis:** Conceptualization (equal); writing – original draft (equal); writing – review and editing (equal). **Shannon Virtue:** Conceptualization (equal); writing – review and editing (equal). **Adam Davey:** Conceptualization (lead); formal analysis (lead); funding acquisition (lead); methodology (lead); software (lead); writing – original draft (equal); writing – review and editing (lead).

## CONFLICT OF INTEREST STATEMENT

Authors report no conflicts.

## ETHICS STATEMENT

The University of Delaware's Institutional Review Board determined that this work was exempt from human subjects considerations (protocol #1998997–1).

## Data Availability

All data reported in this study are publicly available and were obtained from https://www.cdc.gov/nchs/nhis/1997‐2018.htm.
